# On the Many-Body Expansion of an Interaction Energy of Some Supramolecular Halogen-Containing Capsules

**DOI:** 10.3390/molecules26154431

**Published:** 2021-07-22

**Authors:** Jiří Czernek, Jiří Brus

**Affiliations:** Institute of Macromolecular Chemistry, Czech Academy of Sciences, Heyrovsky Square, 16206 Prague, Czech Republic; brus@imc.cas.cz

**Keywords:** non-covalent interactions, supramolecular capsules, interaction energy, DFT

## Abstract

A tetramer model was investigated of a remarkably stable iodine-containing supramolecular capsule that was most recently characterized by other authors, who described emergent features of the capsule’s formation. In an attempt to address the surprising fact that no strong pair-wise interactions between any of the respective components were experimentally detected in condensed phases, the DFT (density-functional theory) computational model was used to decompose the total stabilization energy as a sum of two-, three- and four-body contributions. This model considers complexes formed between either iodine or bromine and the crucial *D_4d_*-symmetric form of octaaryl macrocyclic compound cyclo[8](1,3-(4,6-dimethyl)benzene that is surrounded by arenes of a suitable size, namely, either corannulene or coronene. A significant enthalpic gain associated with the formation of investigated tetramers was revealed. Furthermore, it is shown that the total stabilization of these complexes is dominated by binary interactions. Based on these findings, comments are made regarding the experimentally observed behavior of related multicomponent mixtures.

## 1. Introduction

The molecular self-assembly is one of the most important directions in supramolecular chemistry [1], and a number of applications of synthetic self-assembled architectures were reported (see recent reviews [2,3,4,5,6] and references cited therein). Many multicomponent self-assembled systems were prepared, which were surveyed by Yang et al. [7]. Their association is known to be driven by direct interactions between pairs of the components. Most recently, however, Yang et al. [7] obtained a remarkably stable multicomponent self-assembled capsule (see below), while no pair-wise interactions between its respective subunits were apparent in solution and solid states. This is clearly an important finding, whose significance goes beyond synthetic organic chemistry. It is relevant to crystal engineering [8] and to molecular recognition through non-covalent interactions in general [9], in addition to its possible applications in a preparation of materials with desirable properties, as already described in reference [7]. The structural model of related multicomponent aggregates was thus investigated here. Specifically, high-level quantum chemical calculations were applied in order to estimate the respective contributions to the total stabilization of several four-component systems. Those systems consist of a “shell” formed by two arenes (either corannulenes or coronenes) around the crucial macrocycle of approximately *D_4d_*-symmetric cyclo[8](1,3-(4,6-dimethyl)benzene [10,11] that is abbreviated as CDMB in the following, and of a “core” containing either molecular iodine or molecular bromine (I_2_ or Br_2_). Answers are sought to the following three main questions: (1) How big is the enthalpic gain associated with a formation of the CDMB-containing tetramers? (2) What is the breakdown into two-, three-, and four-body contributions of the total stabilization of those complexes? (3) Are there vast differences in the enthalpic stabilization predicted for different tetramers (containing CDMB together with either two corannulenes or two coronenes and either one I_2_ or one Br_2_ molecule; throughout the article, acronyms Cora and COR are used for corannulene and coronene, respectively)? The presented DFT (density-functional theory) computational results are expected to be useful in exploring the relationship between intermolecular interactions and stability of molecular containers. In particular, they provide context for “emergent behavior” of the iodine-containing capsule most recently described by Yang et al. [7], and thus they contribute to the understanding of factors that govern the self-assembly mechanisms (see the important review article [12]).

## 2. Results and Discussion

**Figure 1:**
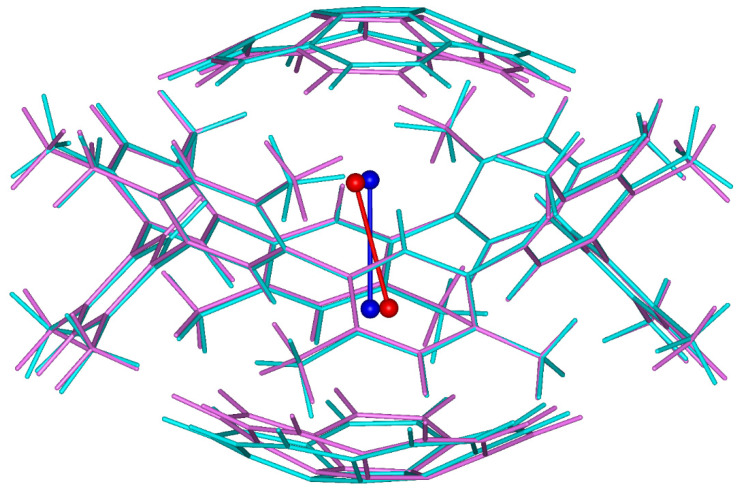
Initial and final geometries of the tetramer discussed in the text. Color coding of the initial/final structures: blue/red for molecular iodine, cyan/magenta for the rest of the atoms.

**Scheme 1:**
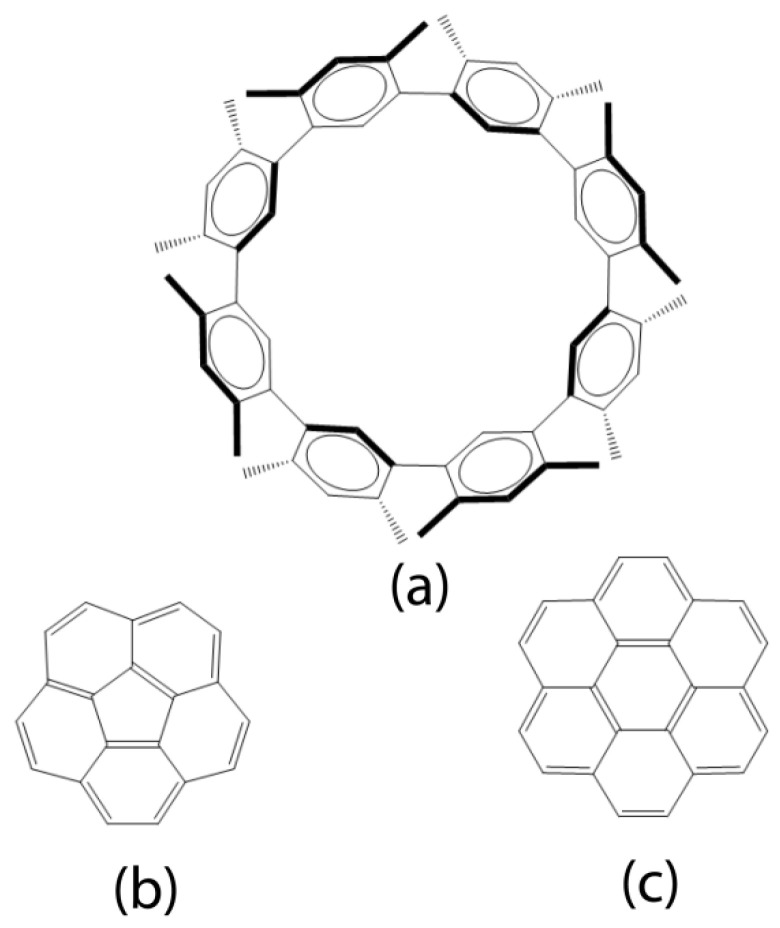
Schematic drawings of *D_4d_*-symmetric cyclo[8](1,3-(4,6-dimethyl)benzene, *C_5v_*-symmetric corranulene, and *D_6h_*-symmetric coronene in panels (**a**), (**b**), and (**c**), respectively.

The tetramer formed by one CDMB molecule with iodine inside and surrounded by two coroannulenes is shown in Figure 1 (see also Scheme 1). Its initial geometry was clipped out from the X-ray diffraction (XRD) structure of a cyclohexane solvate of the iodine-containing capsule [7]. In that structure, whose CCSD refcode is IKEZUW, molecular iodine can be seen to be parallel to the *C_4_* rotation axis of neighboring CDMB units. This symmetry was broken in the course of the DFT optimization (the methodology is detailed in Part 3). However, the angle is small between initial and final I_2_ bond vectors, namely, it amounts to 15°. In addition, orientations of the macrocycle and corannulene units are quite similar in the XRD and DFT geometries (see Figure 1).

Table 1 presents the decomposition of many-body contributions to the DFT interaction energy of the tetramer supermolecule, which were obtained on the basis of Equations (1) and (2) that are given in the Materials and Methods section. The $\Delta E_{\mathrm{tetramer}}^{ABCD}$ value is completely dominated by the following terms (their sum amounts to 98.9% of the total interaction energy): the dimers of CDMB and either corannulene (62.6%), the dimers of iodine and either corannulene (22.1%), and the CDMB…I_2_ dimer (14.2%). These values were checked against their counterparts obtained from the RI-MP2/def2-TZVP energies (the resolution-of-the-identity integral approximation to the second-order Møller–Plesset method combined with an application of the triple-zeta valence plus polarization basis set; see Part 3 for details) computed for the same DFT geometry. The MP2-based results are qualitatively similar: in this case the $\Delta E_{\mathrm{tetramer}}^{ABCD}$ value almost exactly (within 1%) amounts to a sum of contributions from dimers of CDMB and corannulenes (58.0%), of iodine and corannulenes (28.3%), and of iodine and CDMB (13.0%), while all the remaining terms are small (see the Supplementary Materials Table S1). Hence, there is practically no cooperativity (in its classical definition [13]) in the enthalpic stabilization of this tetramer. It is an important finding in the context of experiments [7], which did not reveal any strong binary interactions that would lead to a formation of related dimeric complex(es) in condensed phases. It thus seems that in mixtures containing CDMB, corannulene and iodine, cyclohexane solvent molecules played a decisive role in the emergent behavior of this system that was most recently reported by Yang et al. [7] (its components did not crystallize or associate in solution in pair-wise fashion, but together they formed a highly stable capsule). At this point, it is worth bringing an example of the ethanol tetramer, in which many-body effects are crucial for its enthalpic stabilization. Specifically, the trimers account for 21.4% of the $\Delta E_{\mathrm{tetramer}}^{ABCD}$ value (details are provided in the Supplementary Materials, Table S3).

The DFT calculations were also used to search the potential-energy surface of all constituting dimers and trimers to try to locate their global minima and subsequently evaluate thermodynamic parameters. Unfortunately, no minimum was found in the case of CDMB…Cora…I_2_ trimer. Its lowest-energy structure, which is a transition state, is considered below anyway. Results are summarized in Table 2, wherein data denoted as $\Delta E_{\mathrm{isol}}$ refer to the CP corrected interaction energy, while ΔG refers to the Gibbs free energy of formation estimated at T = 298.15 K using a crude model [14]. Of course, it is not possible to immediately compare the interaction energy of isolated dimers with the corresponding $\Delta E_{\mathrm{tetramer}}^{ABCD}\;$of dimers embedded in the tetramer from Table 1, because different geometries and counterpoise procedures were adopted in the respective calculations. Still, it is worth noting that the enthalpic gain during the tetramer formation is high, as $\Delta E_{\mathrm{tetramer}}^{ABCD}\;$exceeds −300 kJ/mol, while the stabilization conferred by CDMB…Cora dimers is around −100 kJ/mol, for Cora…I_2_ is around −40 kJ/mol, etc. An inspection of $\Delta E_{\mathrm{isol}}$ values reveals that the stabilization of trimers is approximately additive (for example, $\Delta E_{\mathrm{isol}}$ of the CDMB…Cora dimer is −96.3 kJ/mol that amounts to 49.1% of the interaction energy of −196.1 kJ/mol of the trimer formed by one CDMB and two corannulenes). Interestingly, a spontaneous formation of all the dimers and trimers at room temperature is predicted on the basis of estimated ΔG data. This can be contrasted with the situation of ethanol tetramer, whose ΔG is +383 kJ/mol due to a large entropic penalty vastly surpassing the enthalpic stabilization (see the Supplementary Materials, Table S4, for details). Results of the present ΔG calculations show that there is a substantial intrinsic tendency of all three components of the capsule (CDMB, corannulene, and I_2_) to associate despite an absence of highly stabilizing contacts (like salt bridges or strong hydrogen bonds) in the studied complexes. This tendency is yet another indication that cyclohexane molecules are crucial for the emergent behavior of the investigated system [7].

It could be of interest to compare the interaction energies obtained for the aforementioned cluster to its several alternatives, which feature different “shell” and/or “core” fragments of theoretical models of the capsule. Table 3 summarizes results for the four tetramers considered here. From among them, the highest binding energy of ca. −288 kJ/mol is found for the structure already described. This binding energy is followed by a value, which is lower by ca. 18 kJ/mol, that pertains to the tetramer containing molecular bromine instead of iodine. Importantly, Yang et al. [7] presented a preliminary characterization of bromine capture from mixtures containing CDMB and corannulene, and so it is likely that a related self-assembled system will be prepared. The binding energies of coronene-containing tetramers are further lowered by additional ca. 13 and 14 kJ/mol for systems with bromine and iodine, respectively (see Table 3). These differences are relatively small, and hence it appears that also coronenes might form a shell part of complexes with CDMB and a host molecule. The decomposition of a total CP interaction energy obtained for the three modified clusters is shown in Table 4. In the same way as in the cluster presented in Table 1, predominant contributions to $\Delta E_{\mathrm{tetramer}}^{ABCD}\;$come from neighboring dimers.

## 3. Computational Methods

The following monomers and their point group symmetry (in parentheses) were considered: I_2_ (*D_∞h_*); Br_2_ (*D_∞h_*); CDMB (*D_4d_*); corannulene, abbreviated as Cora (*C_5v_*); coronene, abbreviated as COR (*D_6h_*); and ethanol (*C_s_*). The isolated dimers and trimers were: CDMB…Cora; CDMB…I_2_; Cora…Cora; Cora…I_2_; Cora…CDMB…Cora; Cora…CDMB…I_2_; and Cora…I_2_…Cora, and were all of *C_1_* symmetry. Of *C_1_* symmetry were also tetramers comprising: CDMB, two Cora molecules, and I_2_; CDMB, two COR molecules, and I_2_; CDMB, two Cora molecules, and Br_2_; and CDMB, two COR molecules, and Br_2_. The investigated ethanol tetramer was of *S_4_* symmetry (it is a cyclic structure comprising monomers in gauche configuration [15]). All these structures were treated at the B3LYP-D3/6-311G** level of quantum chemical theory, that is, by combining the standard Becke’s three-parameter exchange and the Lee–Yang–Parr correlation DFT functionals together with an unmodified D3 empirical dispersion-correction [16] and with the standard all-electron 6-311G** basis set. Their geometries were fully optimized, and their harmonic vibrational analysis was performed to obtain values of the zero-point energy, the vibrational thermal energy, and the entropy of the respective components in order to estimate in a routine way [14] the Gibbs free energy change at 298.15 K accompanying the formation of the aforementioned dimers and trimers that is denoted simply as ΔG. Subsequently, the deformation energy, ΔE(deform), of the monomers was obtained as the energy difference between the monomer embedded in an investigated complex and of an isolated monomer. An influence of scalar relativistic effects upon the interaction energies, ΔE(relat), was approximated at the B3LYP-D3 level while using the Douglas–Kroll–Hess approach combined with the triple-zeta valence plus polarization basis sets of Jorge et al. [17]. The ΔE(deform) and ΔE(relat) data were added to the total counterpoise-corrected (CP) interaction energy [18] $\Delta E_{\mathrm{tetramer}}^{ABCD}$ (see right below), and to the related difference of zero-point energies, ΔE(ZPE), to arrive at a value of the binding energy, $D_{0}$, of investigated tetramers. The CP complexation energies were computed using the site–site function counterpoise method [19]. In the following, respective components of a tetramer are denoted by integers from the {1, 2, 3, 4} set and the indices i, j, k go over them. Additionally, the superscript ABCD is always used to indicate that in this method the basis functions are at all nuclei of an investigated tetramer (the situation could be different if other schemes were used, see reference [20] for a recent discussion). Thus, for instance, $E_{1,2,3,4}^{ABCD}$ symbol designates the energy of a full tetramer; $E_{1}^{ABCD}$ is the energy of a first unit with ghost nuclei in the positions of units 2, 3, and 4; $E_{2,3}^{ABCD}$ is the energy of the dimer comprising units 2 and 3, and ghost nuclei of units 1 and 4, etc. Based on an expansion of the total energy of a cluster containing N particles into one-particle, two-particle, three-particle, … etc. energies [21], the following two equations for the CP interaction energy $\Delta E_{\mathrm{tetramer}}^{ABCD}\;$are formed:

(1) $$\Delta E_{\mathrm{tetramer}}^{ABCD}=\Delta E_{1,2,3,4}^{ABCD}-\overset{4}{\underset{x=1}{\sum }}E_{{\left(\mathrm{monomer}\right)}_{x}}^{ABCD}$$

and

(2) $$\Delta E_{\mathrm{tetramer}}^{ABCD}=\overset{6}{\underset{y=1}{\sum }}E_{{\left(\mathrm{two}-\mathrm{body}\right)}_{y}}^{ABCD}+\overset{4}{\underset{z=1}{\sum }}E_{{\left(\mathrm{three}-\mathrm{body}\right)}_{z}}^{ABCD}+E_{\left(\mathrm{four}-\mathrm{body}\right)}^{ABCD}$$

where

$$\overset{6}{\underset{y=1}{\sum }}E_{{\left(\mathrm{two}-\mathrm{body}\right)}_{y}}^{ABCD}=\overset{3}{\underset{i=1}{\sum }}\overset{4}{\underset{j=i+1}{\sum }}E_{i,j}^{ABCD}-E_{i}^{ABCD}-E_{j}^{ABCD}$$

and

$$\begin{array}{ll} \overset{4}{\underset{z=1}{\sum }}E_{{\left(\mathrm{three}-\mathrm{body}\right)}_{z}}^{ABCD} & =\overset{2}{\underset{i=1}{\sum }}\overset{3}{\underset{j=i+1}{\sum }}\overset{4}{\underset{k=j+1}{\sum }}\left(E_{i,j,k}^{ABCD}-E_{i}^{ABCD}-E_{j}^{ABCD}-E_{k}^{ABCD}\right)-\left(E_{i,j}^{ABCD}-E_{i}^{ABCD}-E_{j}^{ABCD}\right)\\ & -\left(E_{i,k}^{ABCD}-E_{i}^{ABCD}-E_{k}^{ABCD}\right)-\left(E_{j,k}^{ABCD}-E_{j}^{ABCD}-E_{k}^{ABCD}\right). \end{array}$$

The shorthand notation in “(monomer)*_x_*” subscript is used for the corresponding monomers, “(two-body)*_y_*” for dimers together with relevant monomers, and “(three-body)*_z_*” for trimers together with relevant dimers and monomers. The inclusion–exclusion formula [22] was applied in Equation (2) in order to avoid double counting of the respective contributions, as the presented procedure requires only 15 energies to be computed to obtain all terms of Equations (1) and (2) in a general case. It should be noted that this number is lowered if a tetramer is symmetric. Specifically, the number of unique energies is five in the case of the *S_4_*-symmetric ethanol tetramer. They are $E_{1,2,3,4}^{ABCD}$ together with for example $E_{1}^{ABCD}$, $E_{1,2}^{ABCD}$, $E_{1,3}^{ABCD}$, and $E_{1,2,3}^{ABCD}$ (the energy of one of the monomers, one of the neighbor dimers, one of the diagonal dimers, and one of the trimers, respectively).

In addition to the aforementioned calculations on target systems, the B3LYP-D3/6-311G** interaction energies were obtained for a small set of halogen-containing dimers (see the Supporting Materials, Table S2 and Figure S1), whose highly accurate geometries obtained by Hobza and his coworkers [23] were taken from the BEGDB database [24], so as to confirm that this computational approach provides qualitatively correct results for various types of intermolecular interactions involving iodine and bromine sites. Computations were carried out using the Gaussian 16 program package [25]. Default settings were applied except for the interaction energy calculations, which were performed with a tighter integration grid, namely, with “Grid = SuperFine” option of “Integral” parameter. All the B3LYP-D3/6-311G** optimized geometries and total energies are provided in the Supporting Materials.

The B3LYP-D3/6-311G** geometry of the tetramer comprising CDMB, two Cora molecules, and I_2_ was also employed to obtain the interaction energies at the RI-MP2/def2-TZVP level, namely, by using the RI integral approximation [26] and the corresponding orbital [27] and auxiliary [28] basis sets in version 7.1 of the Turbomole software [29].

## 4. Conclusions

Results of the present B3LYP-D3/6-311G** calculations show a strong enthalpic stabilization of molecular iodine inside the CDMB macrocycle surrounded by coronenes, which is in line with the suggestion that those components are in a thermodynamic minimum under the conditions described in reference [7]. The many-body decomposition of the total interaction energy of the tetramer model reveals only negligible contributions from three- and four-body terms. This is an indirect indication of cyclohexane solvent leading to the emergent behavior of the multicomponent mixtures, which was observed experimentally [7]. Tetramers with the changed composition (iodine replaced with bromine, corannulenes replaced with coronenes) are predicted to be considerably stable, too, and thus the corresponding systems are expected to also form supramolecular capsules.

## Figures and Tables

**Table 1:** The breakdown of contributions to $\Delta E_{\mathrm{tetramer}}^{ABCD}$ (in kJ/mol) defined in Equation (2).

Interaction		$\Delta E^{ABCD}$
Two-body	CDMB…Cora′	−96.6
	CDMB…Cora″	−96.8
	CDMB…I_2_	−44.0
	Cora’…Cora″	−1.1
	Cora’…I_2_	−34.1
	Cora”…I_2_	−34.0
	Σ (dimers)	−306.5
Three-body	Cora’…CDMB…Cora”	−0.3
	Cora′…CDMB…I_2_	−2.6
	CDMB…I_2_…Cora″	−2.5
	Cora′…I_2_…Cora″	+2.8
	Σ (trimers)	−2.6
Four-body	Cora’…CDMB…I_2_…Cora”	+0.5
Total	tetramer formation	−308.7

**Table 2:** The energies (in kJ/mol) of the most energetically favorable structures as found during searches of the potential-energy surface. See the text for details.

Complex	$\Delta E_{\mathrm{isol}}$	ΔG
CDMB…Cora	−96.3	−35.3
CDMB…I_2_	−46.3	−20.6
Cora…Cora	−54.2	−7.6
Cora…I_2_	−40.0	−5.4
Cora…CDMB…Cora	−196.1	−70.7
Cora…CDMB…I_2_	−179.0	−72.8
Cora…I_2_…Cora	−91.6	−6.8

**Table 3:** The interaction energy contributions and the total binding energy of the investigated tetramers. All values are in kJ/mol.

Term	System			
	CDMB(Cora)_2_I_2_	CDMB(COR)_2_I_2_	CDMB(Cora)_2_Br_2_	CDMB(COR)_2_Br_2_
$\Delta E_{\mathrm{tetramer}}^{ABCD}$	−308.7	−273.5	−293.6	−278.6
ΔE(ZPE)	16.4	12.5	17.4	18.1
ΔE(deform)	6.7	9.4	10.6	4.6
ΔE(relat)	−2.5	−4.0	−4.4	−1.7
$D_{0}$	−288.1	−255.6	−270.0	−257.5

**Table 4:** The breakdown of contributions to $\Delta E_{\mathrm{tetramer}}^{ABCD}$ (in kJ/mol) of the three investigated tetramers.

Interaction ^1^		System		
		CDMB(COR)_2_I_2_	CDMB(Cora)_2_Br_2_	CDMB(COR)_2_Br_2_
Two-body	A…B′	−94.3	−98.0	−101.1
	A…B″	−95.1	−97.7	−101.0
	A…C	−39.9	−35.1	−31.8
	B′…B″	−0.6	−1.4	−1.0
	B′…C	−23.1	−29.3	−21.4
	B″…C	−23.2	−29.3	−21.3
	Σ (dimers)	−276.2	−290.7	−277.6
Three-body	B′…A…B″	−0.1	−0.5	−1.4
	B′…A…C	−0.3	−2.0	−1.3
	A…C…B″	−0.2	−2.4	−1.2
	B′…C…B″	+4.7	+1.5	+3.1
	Σ (trimers)	+4.1	−3.3	−0.8
Four-body	B′…A…C…B″	−1.4	+0.4	−0.2
Total	tetramer formation	−273.5	−293.6	−278.6

^1^ Designated accordingly A…B′…B″…C.

## Data Availability

The data presented in this study are available in the article and in the supplementary materials.

## Supplementary Materials

The following are available online. “SI.PDF” file containing comparison of the RI-MP2 and DFT results (Table S1); description of the test set of interaction energies (Table S2 and Figure S1); and additional data for the ethanol tetramer (Tables S3 and S4). Optimized coordinates and their total energies in XYZ format files, which have “xyz” suffix and self-explanatory names: I2, Br2, CDMB, Cora, COR, ethanol, CDMB-Cora, CDMB-I2, Cora-Cora, Cora-I2, Cora-CDMB-Cora, Cora-CDMB-I2, Cora-I2-Cora, Cora-CDMB-I2-Cora, Cora-CDMB-Br2-Cora, COR-CDMB-I2-COR, COR-CDMB-Br2-COR, and ethanol_tetramer.
