# Supplementary material for: On the Many-Body Expansion of an Interaction Energy of Some Supramolecular Halogen-Containing Capsules
Source: Molecules. 2021 Jul 22;26(15):4431. doi: 10.3390/molecules26154431 (PMC8347495; doi:10.3390/molecules26154431)
Supplement: Supplementary file 1 [file molecules-26-04431-s001.zip › SI/SI.pdf]

Supplementary Materials to “On the many-body expansion of an interaction energy of some supramolecular halogen-containing capsules” by Czernek & Brus (*Molecules* 2021)

Table of Contents

The comparison of RI-MP2 and DFT results (Table S1) ……………………………. page SI2

The test set of interaction energies (Table S2 and Figure S1) ……………………….. page SI3

Results for the ethanol tetramer (Tables S3 and S4) …………………………………. page SI4

Table S1. The breakdown of contributions to $\Delta E_{\mathrm{tetramer}}^{ABCD}$ (in kJ/mol) of the tetramer comprising CDMB, two Cora molecules, and I_2. See the main text for details.

interaction		ΔE^{ABCD} (B3LYP-D3/6-311G**)	ΔE^{ABCD} (RI-MP2/def2-TZVP)
two-body	CDMB…Cora′	–96.6	–106.2
	CDMB…Cora″	–96.8	–107.2
	CDMB…I_2	–44.0	–47.9
	Cora′…Cora″	–1.1	–1.9
	Cora′…I_2	–34.1	–52.2
	Cora″…I_2	–34.0	–52.2
	Σ (dimers)	–306.5	–367.5
three-body	Cora′…CDMB…Cora″	–0.3	+0.0
	Cora′…CDMB…I_2	–2.6	–1.1
	CDMB…I_2…Cora″	–2.5	–1.1
	Cora′…I_2…Cora″	+2.8	+1.3
	Σ (trimers)	–2.6	–0.8
four-body	Cora′…CDMB…I_2…Cora″	+0.5	+0.5
total	tetramer formation	–308.7	–367.8

Table S2. The interaction energies (in kJ/mol) that are plotted in Figure S1 and show the correct ordering of the present B3LYP-D3/6-311G** results relative to the benchmark CCSD(T)/CBS values, which come from http://www.begdb.org/index.php?action=oneDataset&id=30&state=show&order=ASC&by=name_m&method=, despite expected differences of absolute values (the mean absolute error is 0.52 kcal/mol; the MP2/cc-pVTZ CP geometries were used to obtain both sets of interaction energies).

dimer	computational approach	
	CCSD(T)/CBS	B3LYP-D3/6-311G**
bromomethane...formaldehyde	–7.205	–7.078
iodomethane...formaldehyde	–9.707	–8.964
bromobenzene ..acetone	–10.150	–9.564
iodobenzene...acetone	–14.477	–12.674
bromobenzene...trimethylamine	–15.807	–16.376
iodobenzene...trimethylamine	–24.297	–23.071
bromomethane...benzene	–7.590	–7.677
iodomethane...benzene	–10.389	–10.711

Figure S1. Plot of the interaction energies from the test set described above.

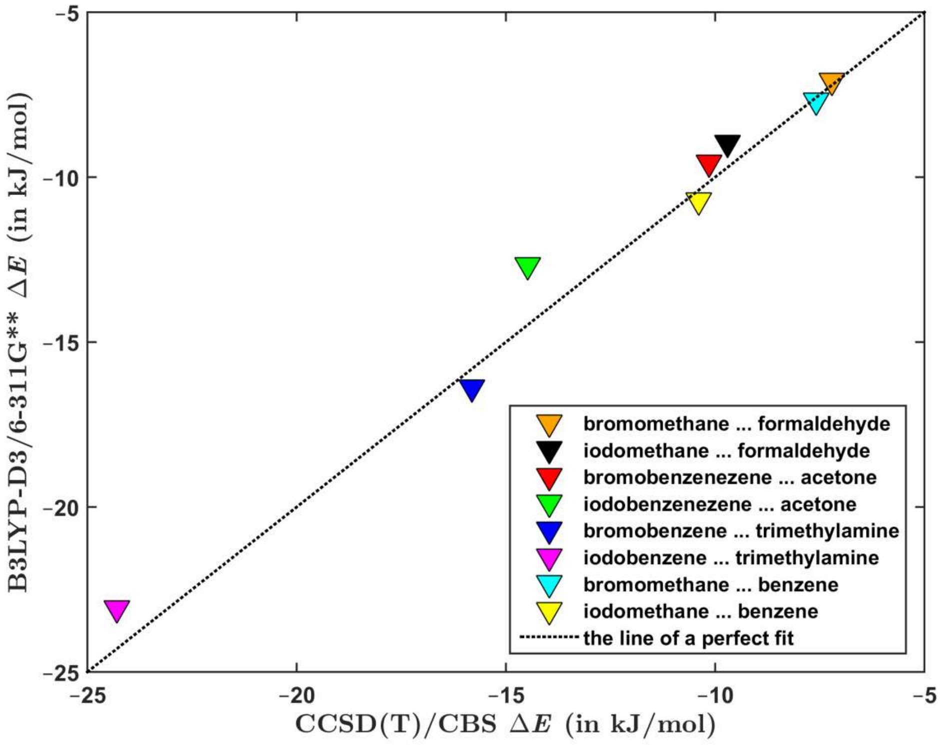

Table S3. The breakdown of contributions to $\Delta E_{\mathrm{tetramer}}^{ABCD}$ (defined in the main text) obtained for the *S₄*-symmetric ethanol tetramer.

interaction		ΔE^{ABCD} value (in kJ/mol)
two-body	each of neighbor dimers	–23.977
	each of diagonal dimers	–9.472
	Σ(dimers)	–114.850
three-body	each of trimers	–7.960
	Σ(trimers)	–31.838
four-body	applying Equations (1) and (2) of the main text	–2.007
total	tetramer formation	–148.695

Table S4. The energetics data at T = 298.15 K related to a formation of the S_4-symmetric ethanol tetramer.

parameter	value (in kJ/mol)
ΔE^{ABCD}	–148.7
$\Delta E(\mathrm{ZPE})$	+21.9
ΔH (includes the vibrational thermal energy; see reference **[14]** of the main text)	–336.4
$T\Delta S$	+719.7
ΔG	+383.3
